# Nursing students’ learning flow, self-efficacy and satisfaction in virtual clinical simulation and clinical case seminar

**DOI:** 10.1186/s12912-023-01621-1

**Published:** 2023-12-01

**Authors:** Hyein Choi, Sunghee H. Tak, Dayeon Lee

**Affiliations:** 1 College of Nursing, Seoul National University, 103 Daehak-ro, Jongno-gu Seoul, 03080 Republic of Korea; 2 Research Institute of Nursing Science, College of Nursing, Seoul National University, 103 Daehak-ro, Jongno-gu Seoul, Republic of Korea

**Keywords:** Clinical education, Virtual simulation, Case seminar, Learning outcome

## Abstract

**Background:**

Virtual clinical simulations and clinical case seminar become widely utilized to address these constraints and help nursing students acquire clinical competencies as the limitations on practicum opportunities have been intensified by the COVID-19 pandemic. The purpose of this study was to examine learning flow, self-efficacy and satisfaction in virtual clinical simulation and clinical case seminar among nursing students.

**Methods:**

A descriptive cross-sectional study was used. Forty-two junior nursing students completed survey questionnaires after participating in computer-based virtual clinical simulation and clinical case seminar, which aimed at acquiring knowledge and care skills in geriatric nursing.

**Results:**

Significant differences in two methods were found in learning flow which included challenge-skill balance (t = -2.24, p < .05) and action-awareness merge (t = -3.32, p < .01). There was no significant difference in learning self-efficacy (t=-1.52, p = .137) and learning satisfaction (t=-0.92, p = .365).

**Conclusions:**

When there’s a mismatch between the perceived challenge and the students’ skill levels, it can hinder the learning process. Therefore, instructors should evaluate the clinical skill levels of their students and make necessary adjustments to the difficulty levels of simulation and clinical case seminar accordingly.

## Background

Clinical practicum is essential for nursing students to apply nursing care and attain the clinical competencies of a registered nurse [1]. The diversity and flexibility of learning methods are critical for both learners and educators [2]. Nonetheless, conventional face-to-face, hands-on clinical experiences in authentic practice settings frequently face constraints or become unfeasible. The limitations on practicum opportunities have been intensified by the COVID-19 pandemic, especially within geriatric settings including geriatric hospitals, senior centers, and nursing homes. Consequently, virtual clinical simulations and clinical case seminar become widely utilized to address these constraints and help nursing students acquire clinical competencies [3, 4].

Due to recent advancements in information technology, virtual reality has become a popular delivery medium for simulation in nursing clinical practicum. Virtual clinical simulation allows learners to experience a variety of audiovisual stimuli and to practice through computer-generated scenarios [5, 6]. Previous studies have shown that virtual clinical simulation is effective for promoting problem-centered thinking ability, learning satisfaction, and nursing performance confidence in students [6–8]. In particular, computer-based virtual clinical simulation allows student nurses to experience clinical situations based on nursing care scenarios through their own computers. Immersive virtual clinical simulations require accessories such as a head-mounted display and a remote control, but computer-based simulations conveniently allow users to practice clinical nursing care experience based on a personal computer or laptop [8, 9]. However, virtual clinical simulations are designed based on structured algorithm-based scenarios and are limited in enabling students to experience various circumstances that may occur in real clinical settings [10].

In contrast, clinical case seminars can draw from real-life patient cases in hospitals, fostering discussions on various approaches to patient care [11, 12]. Clinical case seminars also encourage students to apply theoretical knowledge to actual clinical cases and enhance problem-solving and critical thinking skills, thereby reducing the gap between theory and practice [12]. Previous studies have reported that it is effective for positive learning outcomes such as the development of self-directed learning, critical thinking, and decision-making abilities among nursing students [11–13].

Therefore, given the increasing demand for non-face-to-face practical training, it is crucial to examine two educational methodologies: virtual clinical simulation and clinical case seminars. These methodologies are significant due to their suitability in providing students with a realistic yet indirect experience of clinical nursing practice. However, despite the common use of virtual clinical simulations and clinical case seminars in nursing education, there is limited research exploring the differences in learning flow, self-efficacy, and satisfaction among nursing students. In particular, learning flow is important to measure the effectiveness of an educational method [14, 15]. Learning flow refers to a psychological state in which the learner is completely engaged and actively involved in the learning process, deriving enjoyment and a sense of self-fulfillment [16]. It has been reported as a predictor of clinical reasoning ability in nursing students during practical scenarios [17]. The nine elements of flow include challenge-skill balance, clear goals, unambiguous feedback, the merging of action and awareness, concentration on the present task, sense of control, loss of self-consciousness, transformation of time, and autotelic experiences [18]. The balance between difficulty and self-perceived skill level, or challenge-skill balance, has been particularly emphasized in learning flow. Evidence shows that the most important factor for learning flow is the challenge-skill balance [15]. In other words, learning flow is induced through a balance between the challenge level and the skill level of the student during practice.

In addition, learning self-efficacy means the degree of individual confidence in whether or not the learner can utilize their newly gained knowledge to implement the learning methodology [19, 20]. Learning satisfaction is a positive response to the learning process. Learning satisfaction has been reported to correlate with practical clinical performance among nursing students, indicating that the higher the learning satisfaction, the higher the willingness of students to continue learning [21].

Thus, the purpose of this study is the purpose of this study is to examine the learning flow, learning self-efficacy, and learning satisfaction in two educational methods—virtual clinical simulation and clinical case seminar—among undergraduate nursing students.

## Methods

### Study design

The study used a cross-sectional survey to examine learning flow, self-efficacy and satisfaction in virtual clinical simulation and clinical case seminar among nursing students. The variables included nine elements of learning flow: challenge-skill balance, clear goals, unambiguous feedback, the merging of action and awareness, concentration on the present task, sense of control, loss of self-consciousness, transformation of time, and autotelic experiences. In addition, learning self-efficacy, and learning satisfaction were assessed.

### Setting and participants

Study participants were recruited from junior students attending a nursing college in Seoul, South Korea. Using the G*Power 3.1 program after setting the significance level at 5.0%, the power at 80.0%, and the median effect size at 0.50, a minimum of 34 samples were needed [22]. Forty-two students who participated in both educational methods (virtual clinical simulation and clinical case seminar) completed the survey and were included in the final analysis. The study participants were divided into four groups of 13 to 14 students before receiving the two educational methods. Among the four groups, two had virtual clinical simulation first followed by clinical case seminar, while the other two had the reverse order.

### Educational methods

#### Virtual clinical Simulation

In this study, vSim® for Nursing was utilized for virtual clinical simulation. The vSim® for Nursing is the inaugural online platform connected to the nursing education curriculum. It is a simulation tool originating from the United States, created in 2014 through a partnership involving Wolters Kluwer Health, Laerdal Medical, and the National League for Nursing [23].

Study participants completed two self-directed modules in gerontological nursing. The first module covered geriatric nursing care for an 84-year-old patient who had been hospitalized due to delirium, urinary tract infection, and a fall event. The second module focused on geriatric nursing care for a 65-year-old patient diagnosed with lung cancer, in need of hospice care and family education. Each module included pre-knowledge learning, a pre-quiz, simulation practice, a post-quiz, and feedback, taking approximately 100–150 min to complete. Participants began by reviewing pre-knowledge learning materials and taking pre-quizzes. During simulation practice, they completed patient assessments for delirium, infection, fall risk, and pain, tried out basic nursing skills including vital sign checks or medication administration, interacted with patients and family members, and provided education. After completing simulation practice, they took post-quizzes. The virtual clinical simulation was non-immersive, computer-based, accessible online, and self-guided without any instructor.

Prior to starting the modules, each participant read the user guide and watched a tutorial video explaining how to access and operate the modules of vSim® for Nursing. Study participants used their personal laptops to complete the two modules at home. Upon completion of the two modules, participants were divided into four groups, each comprising 13 to 14 individuals, for a one-hour debriefing session with an instructor. All participants engaged in debriefing sessions facilitated by the same instructor, who was certified in online teaching and doctorally prepared in gerontological nursing education.

#### Clinical case seminar

The clinical case seminar was conducted in face-to-face sessions with 13 to 14 students in a seminar room. It comprised lectures, small group discussions, presentations, and wrap-up sessions. Four experienced clinical nurses currently working in hospitals led these seminars. Each instructor spent approximately 70 min addressing geriatric care issues, including delirium, falls and safety, skin care and pressure ulcers, as well as hospice care and advanced care planning. In total, the clinical case seminar lasted 280 to 300 min.

At the beginning of the clinical case seminar, instructors provided essential information related to each topic and introduced real patient cases from their hospital experiences. Then, study participants, grouped in sets of 3 to 4 people, conducted nursing assessments, diagnoses, care planning, and evaluation plans for their assigned cases. They were encouraged to apply their knowledge and clinical skills, develop care and evaluation plans, and engage in discussions to find solutions and strategies for various disease and symptom-related issues within the cases. The clinical nurses shared their valuable clinical experiences and insights during the clinical case seminar sessions.

### Measurements

#### Learning flow

The Learning Flow Scale for adults, as developed by Kim et al. [24], was utilized to assess learning flow. Learning flow is a psychological state that reflects a learner’s engagement in the learning process [16] and serves as an indicator of the effectiveness of an educational method. This scale is constructed based on nine components of flow as described by Csikszentmihalyi [18], flow levels detailed by Jakson and Marsh [25], and the teaching flow scale developed by Kim et al. [26].

The instrument comprises 29 questions measuring nine sub-factors, including challenge-skill balance, clear goals, unambiguous feedback, action-awareness merge, concentration on the task, sense of control, loss of self-consciousness, transformation of time, and autotelic experiences (Table 1). Each sub-factor is assessed using a 5-point Likert scale. A higher total score across the nine sub-factors indicates a higher level of learning flow. In a prior study by Kim et al. [26], Cronbach’s α for each sub-factor ranged from 0.65 to 0.90. In our study, Cronbach’s α values ranged from 0.87 to 0.97.

**Table 1:** Components of Flow

Components	Definition
Challenge-skills balance	Balance between the challenge of the learning task with the appropriate learner’s skills
Clear goals	Setting clear goals in advance to know exactly what to do
Unambiguous feedback	To be timely aware of whether it is being performed according to the learning objectives
Action-awareness merging	Learners perform learning activities without conscious effort
Concentration on the task at hand	A state of being completely focused on the task without paying attention to anything else
Sense of control	Awareness that learners can perceive their actions
Loss of self-consciousness	A state in which self-awareness is not recognized because of the satisfaction and enjoyment felt in the learning activity itself
Transformation of time	The lack of awareness of time or the disappearance of perception of time during learning
Autotelic experience	Self-independent actions for one’s inner satisfaction, not expectations of profit

In addition, challenge-skill balance was assessed by calculating the difference between perceived challenge and perceived clinical practice skill described by Abuhamdeh and Csikszentmihalyi [27]. Initially, the perceived challenge and perceived skill levels in clinical practice were each rated on a scale from 0 to 100 points. Subsequently, the perceived challenge score was subtracted from the perceived clinical skill score. A negative difference (-1 to -100) indicated that the practice was challenging for students, while a positive difference (1 to 100) indicated that the practice was relatively easy. The closer the difference was to zero, the more balanced the challenge and skill levels were perceived to be.

#### Learning self-efficacy

Learning self-efficacy was assessed using an instrument initially developed by Ayres [28] and later translated by Park and Kweon [29]. The instrument employed a 7-point Likert scale, with higher scores indicating higher levels of learning self-efficacy. The original instrument demonstrated a Cronbach’s α of 0.94 [28], while the translated version had a Cronbach’s α of 0.88 [29]. In our study, Cronbach’s α was 0.99.

#### Learning satisfaction

The Learning Satisfaction Scale, as developed by Shin [30], comprises eight questions designed to assess learning satisfaction. The original tool demonstrated a Cronbach’s α of 0.94. In our study, a Likert scale ranging from one (strongly disagree) to five (strongly agree) was used. The Cronbach’s α was 0.97.

### Data analysis

General characteristics of participants were analyzed using descriptive statistics and frequency analysis. Paired t-tests were conducted to compare learning flow, learning self-efficacy, and learning satisfaction between virtual clinical simulation and clinical case seminar. Data were analyzed using the IBM SPSS 25.0 program for Windows.

### Data collection

After obtaining IRB approval, students enrolled in the gerontological nursing course were invited to participate in the study. Recruitment notices and online survey URLs were distributed via the university communication network to the email addresses of students who had completed both the virtual clinical simulation and the clinical case seminar.

First, students needed to access to the URL site and complete the online consent form. Upon completing this step, they were able to initiate the survey. Study participants completed both the virtual clinical simulation and clinical case seminar within a maximum of four days, with no more than a two-day gap between the two components. They were asked to complete the survey immediately after completing both educational methods. Data were collected only from those who provided their consent and successfully completed the questionnaires through the survey URL.

### Ethical considerations

This study was approved by the Institutional Review Board (IRB No. 210X/XXX-002). The data was collected through an online survey conducted between September 2nd, 2021 and October 14th, 2021. Informed written consent was obtained from the participants. Through the survey URL, participants were asked to confirm their willingness to participate in the research. They could proceed with the questionnaire only after their consent by checking the agreement box. The consent form online explicitly assured that personal information would remain anonymous and that participants retained the freedom to withdraw from the study at any time. Research assistants were available to address questions and provide clarifications via phone or in person as needed. The survey questionnaire online did not request any personally identifiable information. The collected data were maintained in a non-identifiable format and securely stored on the computer of two researchers. This storage involved a double-lock system that included both computer and document safeguards.

## Results

The participants had a mean age of 22 years (SD = 1.82, range 20–26). Among the participants, individuals who were 21 years old accounted for the largest proportion, comprising 38% of the total. The majority of participants were women, accounting for 81%. Approximately 62% of the participants did not have previous experience using virtual reality devices in their daily lives.

Table 2 presents learning flow, learning self-efficacy and learning satisfaction of two educational methods. Regarding the virtual clinical simulation, the average score for the overall learning flow was 2.95. Among the sub-factors of learning flow, the loss of self-consciousness was 2.08, while challenge-skill balance averaged 3.77. The difference between perceived challenge and perceived clinical practice skill was 21.79, indicating that participants in the virtual setting perceived a relatively high challenge compared to their clinical competence. The mean score for learning self-efficacy was 5.01, indicating a moderate to high level of self-efficacy in the virtual learning context. Learning satisfaction averaged 3.71, suggesting a moderate to high level of satisfaction among participants in the virtual clinical simulation.

**Table 2:** Learning Flow, Learning Satisfaction, Learning Self-Efficacy

Variables	Virtual Clinical Simulation	Clinical Case Seminar	t	*p*
	M(SD)	M(SD)		
Learning flow				
Challenge-skills balance	3.77(0.78)	3.96(0.58)	-2.24	0.031
Clear goals	3.24(1.04)	3.26(0.88)	-0.23	0.819
Unambiguous feedback	3.39(1.05)	3.39(1.05)	-1.68	0.101
Action-awareness merging	2.98(1.08)	3.31(0.99)	-3.32	0.002
Concentration on the task at hand	2.86(1.03)	2.92(0.90)	-0.60	0.551
Sense of control	2.92(1.03)	2.87(0.87)	0.47	0.641
Loss of self-consciousness	2.08(0.82)	2.05(0.70)	0.38	0.705
Transformation of time	2.63(1.12)	2.52(1.06)	1.12	0.270
Autotelic experience	2.76(1.08)	2.92(0.96)	-1.38	0.174
Total score	2.95(0.85)	3.04(0.67)	-1.16	0.250
The difference between perceived challenge and perceived clinical practice skill	21.79(23.89)	-22.14(26.00)	-7.10	< 0.001
Learning self-efficacy	5.01(1.49)	5.27(1.29)	-1.52	0.124
Learning satisfaction	3.71(1.01)	3.82(0.85)	-0.92	0.365

On the other hand, the overall score for learning flow in the clinical case seminar was 3.04 on average. Among the sub-factors of learning flow, the loss of self-consciousness was 2.05, while challenge-skill balance averaged 3.96. The difference between perceived challenge and perceived clinical practice skill was − 22.14, suggesting that participants in the clinical case seminar perceived a challenge level significantly lower than their clinical competence. The mean score for learning self-efficacy was 5.27, indicating a strong sense of self-efficacy in the case-based learning context. The score for learning satisfaction averaged 3.82, suggesting a high level of satisfaction among participants in the clinical case seminar.

When comparing virtual clinical simulation and clinical case seminar, significant differences were observed in two components of learning flow: challenge-skill balance (t = -2.24; p = .031) and action-awareness merging (t = -3.32; p = .002). Participants perceived a better balance between the challenge of the learning task and their skills, as well as performed learning activities without conscious effort in the clinical case seminar compared to the virtual clinical simulation. In particular, the differences between perceived challenge and perceived skills were significant (t = -7.10, p < .001) between the two educational methods. The virtual clinical simulation was found to be challenging for the students, with a mean score of -21.79 (SD = 23.89). On the other hand, the clinical case seminar revealed that the perceived skill level in learning was higher than the perceived challenge level, with a mean score of 22.14 (SD = 26.00). However, there was no significant difference in the total score of learning flow (t = -1.16 and *p* = .252) between virtual clinical simulation and clinical case seminar. There were no significant differences in learning self-efficacy (t = -1.52, p = .137) and learning satisfaction (t = -0.92, p = .365) between the two learning methods.

## Discussion

The findings of the study offer valuable insights in the differences in learning flow between virtual clinical simulation and clinical case seminar and help educators make informed decisions regarding instructional strategies in order to enhance the effectiveness of learning. The findings indicated that there is an imbalance between the levels of challenge and skill due to a high level of difficulty in the virtual clinical simulation. Clinical case seminar, in comparison to virtual clinical simulation, demonstrated significantly higher scores for challenge-skill balance and action-awareness merging. The difficulty level of clinical case seminar was effectively balanced by students’ self-perceived clinical skill, indicating that students accomplished their learning without conscious effort.

Previous research has highlighted challenge-skill balance as one of the key factors influencing learning flow [31]. If the perceived challenge level exceeds the learner’s perceived skill level, learning flow may be disrupted. Similarly, if the perceived challenge level falls below the learner’s perceived skill level, learning flow may also be disturbed. Learners perceive a greater sense of balance between challenge and skill levels when engaging with learning that feels relatively easy, as opposed to when engagement feels difficult. In this study, virtual clinical simulation required students’ communication skills with older adults in order to complete the modules of gerontological nursing. Evidence indicates that students often experience difficulties in communicating with older adults [32].

In addition, it is speculated that the students’ language proficiency in English as a second language might have influenced their perception of the difficulty in virtual clinical simulation because it was offered only in English. Communication and language factors need to be considered in the development of virtual clinical simulation programs [32]. To promote a state of learning flow and enhance the effectiveness of learning, it is essential to consider the learner’s self-perceived skill level and establish a suitable level of learning difficulty [33].

In this study, more than half of the participants had no prior experience with virtual reality devices. Because the virtual clinical simulation was non-immersive and computer-based, no virtual reality devices were used in this study. Study participants did not experience any difficulties with the use of virtual reality devices. Previous research has reported that individuals with prior experience in virtual reality devices tend to exhibit a more favorable attitude when embracing virtual reality as a learning method [34]. It’s important to note that there is a lack of research in the existing literature regarding the impact of virtual reality device usage in other domains on the perception of difficulty in the context of learning. Consequently, further investigation is warranted to gain a comprehensive understanding of the relationship between prior device usage in diverse contexts and how it influences the perception of learning difficulty.

Previous research has yielded inconsistent findings regarding learning self-efficacy, which appear to vary depending on the learning methods employed, subject topics, and the specific learning processes involved [35]. Previous studies show that a significant increase in self-efficacy levels following immersive virtual reality simulations with head-mounted displays [8–10]. Similarly, evidence indicates that learning self-efficacy significantly increased when the case seminar method was applied to general lecture subjects [11, 12]. On the contrary, Lee and Noh [36] found that a blended-learning hybrid approach with case seminar did not result in a significant increase in learning self-efficacy. In this study, the mean score of learning self-efficacy for both methods was 5 or higher, indicating a high level of self-efficacy. Further research is needed to gain a deeper understanding of the factors influencing learning self-efficacy.

Evidence indicates a high level of students’ learning satisfaction in both virtual clinical simulation and clinical case seminar. A meta-analysis examining the effectiveness of virtual clinical simulation showed that 10 out of 12 randomized controlled trials reported high levels of learning satisfaction [37]. In addition, evidence shows that students express high satisfaction with nursing skill training through case-based lectures [38]. In this study, the mean scores for learning satisfaction in both virtual clinical simulation and clinical case seminar were relatively high although there were no significant differences in learning satisfaction between the two educational methods.

It is important to note that virtual clinical simulation used in this study was non-immersive. Realism differs from immersive or semi-immersive or non-immersive virtual reality programs [39, 40]. According to a study conducted by Kwon [38], immersive ones offer learners a simulated reality that enhances immersion and aids in learning achievement. Further research is required to investigate variations in learning outcomes across these diverse virtual simulation methods, taking into account the varying levels of immersion and realism they provide. As technological advancements continue, it is important to continuously explore and incorporate innovative approaches to clinical education.

The limited generalizability of the findings in this study, attributed to its single-institution setting and small sample size, necessitates further investigation. A longitudinal design with a large sample size drawn from multiple institutions is recommended to enhance external validity and gain a comprehensive understanding of the effects and applicability of learning methods across diverse educational contexts. Moreover, since the study participants were asked to complete survey questionnaires about two educational methods after participating in both, the order of educational methods may have influenced the study outcomes. In this study, the influence may be lessened because participants were divided into four groups, with two experiencing virtual clinical simulation first followed by a clinical case seminar, and the other two having the reverse order. Future research is needed to examine the effects of each method on learning outcomes in an experimental design with pre-post tests.

## Conclusion

This study examined the learning flow, learning self-efficacy and learning satisfaction in virtual clinical simulation and clinical case seminar. Students perceived virtual clinical simulations as more challenging than clinical case seminars. In both learning methods, it was confirmed that learning self-efficacy and satisfaction were relatively high, with no significant difference observed between the two methods.

The findings suggest that students perceive a significant challenge-skill imbalance in virtual clinical simulation, in comparison to clinical case seminar, despite both covering similar topics and content in geriatric nursing. Therefore, it is essential to assess students’ skill levels to ensure an appropriate level of difficulty that aligns with their abilities. Furthermore, the distinctive characteristics of each educational method should be considered in the development of educational programs. The findings suggest that students perceive a significant challenge-skill imbalance in virtual clinical simulation, in comparison to clinical case seminar, despite both covering similar topics and content in geriatric nursing. Therefore, it is essential to assess students’ skill levels to ensure an appropriate level of difficulty that aligns with their abilities. Furthermore, the distinctive characteristics of each educational method should be considered in the development of educational programs.

## Data Availability

The datasets used and/or analyzed during the current study are available from the corresponding author on reasonable request.
